# Understanding Organisational Ability and Self-Regulation in Children with Developmental Coordination Disorder

**DOI:** 10.1007/s40474-018-0129-2

**Published:** 2018-01-23

**Authors:** Dido Green, Sally Payne

**Affiliations:** 1 School of Health and Welfare, Jönköping University, Jönköping, Sweden; 2 Heart of England Foundation NHS Trust, Birmingham, UK

**Keywords:** DCD, Praxis, Organisation, Emotional regulation, Mechanisms, Participation

## Abstract

**Purpose of the Review:**

This paper examines physical and emotional coherence in young people with developmental coordination disorder (DCD). Specifically, the transactional relationship between motor and non-motor/executive dysfunction in children with DCD and its impact on psychosocial functioning is explored.

**Recent Findings:**

This paper integrates the subjective reported experiences of young people with DCD with objective measurements and findings from neuroimaging studies.

**Summary:**

Consideration of the transactional relationship between the person, the activity and the environment, whether these factors be physical, social, attitudinal or virtual, will be fundamental to our understanding of the mechanisms underpinning organisational and emotional issues presenting in DCD. Integrating the experiences of young people with research evidence will be essential to improve outcomes for young people with DCD in clinical practice.

## Introduction

Children with movement difficulties that impact on functional skills, specifically activities and participation involving motor skills, are categorised as having Developmental Coordination Disorder (DCD) when these difficulties cannot be attributed to age, intellectual disability, experience or known neurological disorder [1]. The diagnosis of DCD has emerged more recently from previous labels, some of which have been subsumed under this overarching term to include concepts of motor learning, motor memory and motor planning.

In this latter respect, the terms dyspraxia or developmental dyspraxia have been used at times synonymously or interchangeably with DCD to capture the phenomenon of ‘organisation of movement’ which involves the process of forming ideas, motor planning and movement execution that can be observed in DCD. More recently the term ‘dyspraxia’ has become associated with other neurodevelopmental disorders especially autism spectrum disorder, and linked to gesture production [2–5]. While an International Consensus for the management of DCD does not recommend this term as it recognises it is not equivalent to DCD, individuals with the condition prefer to use the label of dyspraxia to define their condition or describe themselves [6–8]. Many children and young people nevertheless present with functional difficulties that reflect problems in organisation of behaviour and actions which at face value are not predominately motoric in nature, e.g. organisation of materials on a desktop to prevent them from falling to the floor. These organisational difficulties can impact more on day to day life over time than poor motor coordination.

Additionally, co-occurring mental health difficulties have been identified in people with DCD including internalising problems such as depression and anxiety and externalising behaviours such as Attention Deficit Hyperactivity Disorder and also social impairments [9–11]. These psychosocial problems may persist into adulthood and have significant impacts on adaptive behaviour and mental health outcomes [12–14]. What remains unclear is the extent to which psychosocial issues in DCD are integral features or experiential consequences of a person’s coordination and associated difficulties.

This paper will therefore examine physical and emotional coherence in DCD, including the relationship between thinking, planning, doing and feeling in DCD. New findings regarding executive functions and emotional regulation will be examined, with a focus on the transactional influences of personal, contextual and task-specific factors on behaviour and participation. Examples drawn from a recent qualitative study with teenagers with DCD will be used to contextualise the findings.

## Presentation of Organisational Deficits

Consideration of DCD as more than just a movement difficulty has been evidenced by a number of researchers [15]. The advent of more ecologically valid assessments reflecting ‘real-life’ contexts has highlighted the broader deficits and impact of movement problems of individuals with DCD. In this respect, poor organisation of body movements in relation to space has been shown to impact on capacity to move through gaps, navigate obstacles, and manage daily tasks such as buttering bread or drying oneself after a shower or bath [16–18]. In particular, being able to ride a bicycle is a valued activity for children with DCD in some cultures, but presents additional challenges for these children which are not just due to their difficulties mastering the required motor skills but also due to organisational and planning problems affecting capacity to navigate, particularly over different terrains [19].

Difficulties in learning new motor tasks continue to challenge individuals through to adulthood. Problems may manifest in recording messages or taking notes, preparing meals, packing a suitcase, managing money and many other common daily activities across home, work and community environments [15]. Learning techniques to cope with a new job can be particularly difficult for adults with motor deficits [20]. Driving skills, such as regulating speed and coping with distraction, distance perception and slow reaction times have all been shown to be compromised in young adults with DCD and impact on their capacity to avoid hazards [21].

Qualitative analysis of interviews of teenagers with DCD epitomise some of the organisational difficulties and consequent frustration experienced when performing typical daily tasks. For example, one teenager described his difficulty organising materials in his workspace: “Say I’m eating my breakfast ... and I’ve got the butter open there where I’m going to put my elbow, and the jam there, all in the way” (Billy, aged 13). Billy was frustrated at his inability to organise his breakfast things and embarrassed at the visible signs of his poor coordination (food marks on his clothing). Some children with DCD work hard to manage their poor organisational skills: “Organisation, I haven’t really got any. I don’t sort my school stuff out, I just keep it all in my bag. I carry all my school books around with me” (David, aged 15). An unintended consequence of David’s poor organisation skills however, was the extra time needed to find what he was looking for in his school bag and the additional weight in the bags he carried around all day has implications for back-care (Quotes from Payne 2015 [22]).

In a study exploring the lived experience of teenagers with DCD, participants described their lives as ‘hard work’ because of the extra effort required to master and perform everyday motor tasks and the extra cognitive effort required to organise themselves, their time and their equipment [22]. DCD was for these teenagers, not just a physical construct. Moreover, the combination of motor and non-motor difficulties experienced by young people with DCD impacted on their emotional well-being and social participation.

## Self-Regulation and Resilience in DCD

Children with DCD have been shown to be at increased risk of psychosocial problems affecting their quality of life and participation or engagement in activities at home, at school and in social settings [9, 11, 23–25]. Although more limited, emerging evidence indicates that the association between DCD and poor mental health persists into adolescence [12, 26] and adulthood [13, 27, 28].

Children and adolescents with DCD are at increased risk of both internalising (anxiety, depression) and externalising (e.g. ADHD) behaviours [9, 12, 25, 29–31]. Frustration at their inability to perform activities that others manage to easily accomplish contributes to children’s sense of inadequacy: “It’s frustrating when everyone else can do it straight away, then when I try and draw a cube or something it turns out really weird” (George, aged 13). Children with DCD often express anxiety about things that might happen because of their motor and organisational difficulties: “I always think I’ve forgotten something and something could go wrong. It’s the ‘what if’ thing in my mind” (George, aged 14); and, when faced with activities they have found challenging previously “I don’t feel comfortable using the woodwork equipment at school because it’s sharp and I’m just scared to use it” (David, aged 13) [22]. Parents in a study by Pratt and Hill [30] also reported high levels of ‘panic anxiety’ amongst children with DCD when faced with new or challenging situations or activities.

Research indicates that young people with DCD are generally accurate in their perceptions of motor competence [32] with unfavourable comparisons to their peers leading to low self-esteem and poor self-worth [27, 28]. Poor self-efficacy for motor skills affects the motivation of children with DCD to engage in social and physical activities [24]. Motivation to participate is also influenced by the context in which activities take place: “If it was an activity with people that I didn’t know particularly well, then I wouldn’t want to get involved in case I got it wrong” (Billy, aged 13) [22]. Children with DCD are therefore more likely to engage in fewer social activities than their peers and to choose activities that are quieter and more socially isolated than those chosen by more physical able peers [33]. This is of concern as social isolation is linked to poorer mental health.

Avoidance of physical activities is also reported to be common in children with DCD who may shun situations in which their difficulties might be exposed [25, 34, 35]. While studies suggest that participation in physical activities has a beneficial effect for children’s mental health [36], this may not be the case for children with DCD whose feelings of inadequacy may be reinforced through negative interactions with peers [37].

Recently, gender has been suggested as a factor influencing emotional well-being in young people with DCD. While a previous study by Sigurdsson et al. in 2002 [13] reported higher levels of anxiety in adolescent males compared to females with motor difficulties, a recent study by Harrowell and colleagues [12] showed that females with DCD aged 16–18 years were more likely to report emotional difficulties and depressive symptoms compared to age and gender matched controls. This supports the findings of a survey by The Dyspraxia Foundation in 2015 which revealed that girls were typically not diagnosed, on average, until aged 22 years when their previous coping strategies were no longer effective, leading them to seek support for their coordination, organisational and mental health difficulties [38].

Cairney and colleagues [9] put forward the Environmental Stress Hypothesis as a possible explanation for the increase in mood disorders among children with DCD, with secondary psychosocial consequences of the primary motor disorder having a cumulative negative impact on self-esteem and mental health. Factors such as the context in which activities take place [37], fitness and obesity [39]and peer victimisation [40] all impact on the participation and emotional well-being of children with DCD. Psychosocial well-being is not necessarily linked to the severity of a child’s motor impairments [11, 33] with self-esteem, the presence of co-occurring difficulties, bullying and social communication difficulties being important mediating factors [25].

## Mechanisms Underpinning Organisation of Behaviour and Emotion in DCD

**Fig. 1:**
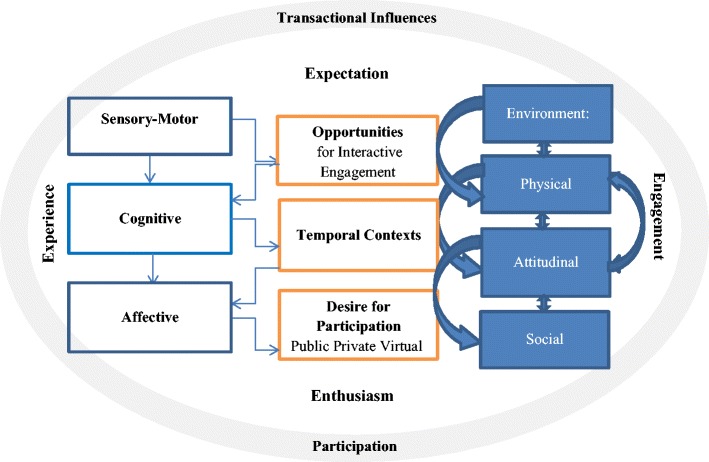
A transactional framework to capture the dynamic interactions between the person, participatory elements and environmental influences

The complexity and heterogeneity in DCD presents challenges in attempts to understand the mechanisms underpinning motor skills, let alone how these relate to organisation of behaviour and emotion. Building on the Causal Model of Developmental Disorders outlined by Morton [41] and developed by Green et al. [42] for children with DCD, a new framework will be presented to consider participatory contexts that reflect the fluid and transactional nature of interactions between the person, the activity and environmental interfaces. This model builds on the evidence reflecting the iterative process of learning and experience (objective and subjective) and its implications for performance and well-being. In this respect, the context in which behavioural organisation and emotional experience occurs is amorphous and includes multiple dynamically interacting variables. A behaviour defined as ‘poorly organised’ in one context may ‘fit’ or be appropriate in a different place, time or social group. The context for performance, including temporal elements, is essential to the understanding of the setting for behaviour and emotional experiences [43]. The transactional framework set out by King and colleagues [43] considers the facilitative, resiliency and socialisation processes that are contextualised by the experiences of the situated person. Consideration of not just the present, but how perceptions of the present have been built from experiences (including effort and success) will then influence expectations, enthusiasm (motivation) and engagement to drive goal-directed behaviours [44]. Incorporation of the temporal elements of the person (subjective and objective) and activity within their environments (physical, social or virtual) will be fundamental to our understanding of the mechanisms underpinning organisational and emotional issues presenting in DCD (See Fig. 1).

The interaction between functional abilities and task performance, including organisational capacities, has been shown to influence caregiver perceptions of environmental supports at home, with these environmental supports impacting on children’s participation in home-based activities during early childhood [45]. The extent to which opportunities to engage in complex tasks, to develop skills and self-competencies, may therefore be restricted early on when children demonstrate movement impairments. Limitations to self-efficacy are reported for children with DCD as young as 5 years [24], and higher levels of bullying and victimisation also reported in childhood [40, 46], exacerbating the negative personal and environmental experiences of individuals with movement problems. The Environmental Stress Hypothesis [9, 47] provides a framework for understanding the association between motor skill performance, the influence of environmental ‘challenges’ (including bullying, limitations to functional performance and poor self-concept) and internalising problems including anxiety and depression. Environmental risks for DCD, particularly low-income/socio economic backgrounds and low birth weight/low gestational age have also been identified as risks for poor emotional adjustment and mental health problems [48]. The interaction between these environmental and psychosocial contexts has implications for access to facilitative physical and attitudinal environmental supports and development of self-competence and resilience.

Stepping back from the macro level of influences on opportunities for skill acquisition, at the personal level there are a number of studies reporting weaknesses in cognitive processes (i.e. executive function; EF) which persist into early adulthood [49]. Deficits in cognitive processes have been linked to impaired planning and organisation in daily life and shown to be independent of the attention problems commonly co-occurring in DCD. Planning problems have also been considered in a study examining anticipatory adjustments to navigate through environmental obstacles (gaps) in which adults with DCD were found to have developed a different adaptive strategy to account for movement problems and avoid collisions [16].

Deficits in EF processes have also been shown to impact on academic performance including concentration and memory [28, 49]. Others have considered cognitive processes and the impact of increasing cognitive effort/cognitive load on motor performance from the perspective of a lack of automisation in motor skill production: “the automisation deficit hypothesis” [50]. Difficulties in EF have also been reported with respect to dual task control, reflecting the effort of multi-tasking and consequent impact on performance and behaviour [51, 52].

Weaknesses in cognitive processes in DCD are consistent with neural accounts for the deficits occurring in DCD when utilising advanced magnetic resonance imaging (MRI) or electroencephalogry (EEG) or transcranial ultrasound [53]. These studies have shown under-activation across a number of key networks involved in motor control and movement execution motor-related tracts including cerebellar peduncles, thalamic radiations, corpus callosum, and corticobulbar and corticospinal tracts [53]. Reduced cortical thickness, particularly in the right anterior temporal pole, has also been identified [54]. Importantly, brain areas involved in anticipatory motor control and error correction (which fits an internal modelling deficit account for DCD) [55, 56] and the mirror neurone system (which has been linked to imitation and potentially ‘praxis’) [57] have been implicated in DCD [53]. White matter connections with reduced fractional anisotropy (FA) of somatosensory tracts, especially, or the retro-lenticular limb of the internal capsule and posterior thalamic radiation, have been associated with reduced visual-motor control [58]. Zwicker et al. [59] reported white matter abnormalities in the posterior thalamic radiation and also cerebellar peduncles for people with DCD, linked to motor learning. Imitation deficits have been found in DCD alongside evidence of reduced activation in brain regions associated with the mirror neurone system (including precentral gyrus, inferior frontal gyrus, and cingulate, as well as lowered activation in the pars opercularis) when children with DCD observed actions [60]. These findings are consistent with the imitation and gesture difficulties influencing motor learning (copying) as well as the social deficits seen in these children [61] and provide some support for these underlying areas contributing to various aspects of the impairments in movement organisation demonstrated in DCD.

**Fig. 2:**
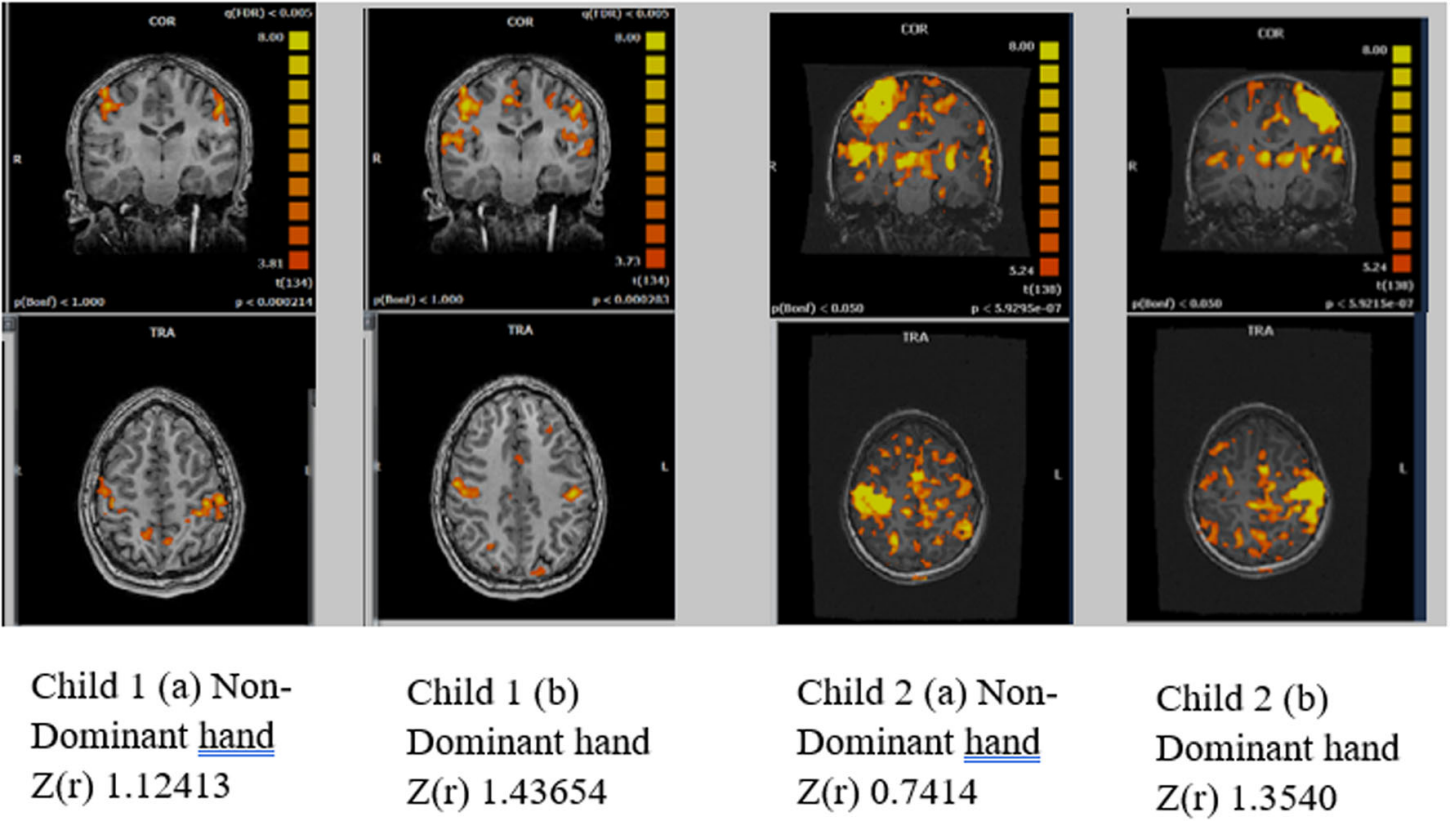
fcMRI seeded from active and passive motor conditions of children with movement impairments with and without organisational and emotional difficulties. Functional connectivity maps based on a seed regions defined from active motor task in dominant and non/dominant sensori-motor cortex. Child 1: male 14 years 3 months, with very mild right hemiparesis due to CP, without behavioural or emotional deficits. Child 2: male 10 years 11 months, with coordination impairment accompanied with behavioural organisational problems and emotional lability.

There have also been reports of weaker segregation and specificity of neural networking and the visuomotor deficits seen in DCD [62]. This is reflected in findings of distributed connectivity in children with movement impairments impacting on behaviour and emotional regulation [63]. Figure 2 illustrates the connectivity profiles from functional connectivity resting state MRI (fcMRI; an indirect measure of brain connectivity through blood oxygenation level-dependent MRI signal) in two children with movement impairments: child 1, having no organisational or emotional problems, with a very mild hemiparesis due to cerebral palsy (CP), but with hand function in the typical range and clear motor network pathways; child 2 with movement impairments consistent with DCD along with considerable functional deficits in organisation, planning and emotional lability and obsessive behaviours and showing multiple and dispersed connectivity from the seeded motor area. The seed region was extracted from the sensori-motor cortex based on an active motor task (see Weinstein et al. for protocol) [64]. The lack of specificity and coherence of the motor network evident in child 2, in which many areas/networks are correlated with the motor seed region, is consistent with the disperse connectivity profiles of language areas reported in individuals with dyslexia [65].

## Facilitators and Barriers to Participation in DCD

Studies and the experience of children with DCD indicate that their motivation and participation in activities is affected not only by the severity of their coordination and organisational difficulties, but also by the context in which activities take place. Children may be less motivated to engage in activities when the risk of exposing their coordination difficulties is high and where their performance impacts on others: “I get anxious about how people will look at me, how I look and stuff at school” (George aged 14) [22]. Identifying situations where participation is valued more highly than performance and where children with DCD perceive there is a good match between performance expectations and their own abilities will therefore support children’s psychosocial well-being, build self-efficacy and encourage their participation.

Social interactions have an important influence on the participation of children with DCD with negative comments having a cumulative impact on self-esteem: “If someone said to me, like a teacher, about my handwriting I would just be less confident for the rest of the day” (George aged 14) [22]. By contrast, children who feel understood and supported by adults are more likely to try new things and to persevere when they encounter challenges. Parents and teachers can support children with DCD by understanding their strengths and difficulties and by adapting and modifying activities to enable their performance. The partnering for change model and M.A.T.C.H. [66] approach encourages teachers to enable children’s participation and achievement by Modifying the task, Altering their expectations, Teaching strategies, Changing the environment and Helping children feel understood and supported. The parent coaching model [67] is used by therapists to help parents recognise barriers and supports to their children’s participation and developing solutions that enable their success. The PREP protocol (Pathways and Resources for Engagement and Participation) [68] is a new strengths-based approach for children with physical disabilities that could be applied to children with DCD, that helps young people and their parents consider how to remove environmental barriers to participation in community-based leisure activities. These intervention approaches are aimed at overcoming the barriers to participation that may be due to motor coordination difficulties and or personal organisational and or psychosocial problems individuals with DCD encounter on a daily basis.

New evidence reflects the benefits of self-management training and coaching in children with DCD through programmes such as the Cognitive Orientation to daily Occupational Performance (COOP Approach; delivered in a group setting) [69]. Self-management approaches that enable individuals to take greater responsibility for their performance and participation are also recommended for teenagers with DCD [8, 26]. Effective self-management programmes help young people to better understand their strengths and difficulties and to identify and apply strategies that support their performance in ecologically valid contexts. Project T.E.A.M. (Teens Making Environment and Activity Modifications) is a self-management programme developed for young people with developmental difficulties and encourages individuals to identify environmental barriers and supports to their participation and provides them with the confidence and skills to request the reasonable adjustments that enable their performance [70].

## Conclusion

Clinical and research indications reflect an overlap between the organisational difficulties experienced by individuals with DCD, particularly planning and activity execution and emotional vulnerability that cannot be explained by the degree of motor impairment. The severity of a person’s motor deficits may be less important for a person’s well-being and participation than the individual’s experience and perceptions of these deficits. A transactional framework has been introduced to capture the experience and perceptions of individuals and how these may be influenced by the contexts in which activities take place and their interactions with others.

Evidence from research and clinical practice indicates that DCD is not just a physical construct. Although co-occurring mental health difficulties have been identified in many people with DCD, there is increasing evidence reflecting deficits in executive functions, behavioural organisation and emotional regulation that extend beyond the motor impairments and which are independent of additional diagnoses. Researchers and clinicians need to consider and address non-motor factors if interventions are to be effective in improving personal, academic, emotional and social outcomes for people with DCD. Examining all these factors (motor, organisational/executive functioning, contextual and social) will help us understand differences in the performance and participation of individuals with DCD in real-life situations and develop interventions that improve the quality of life and life satisfaction of people with DCD.

Multiple methodologies and new theoretical perspectives are therefore needed for a comprehensive framework that can incorporate individual as well as contextual factors into the understanding of the causes and mechanisms of DCD and impacts on performance and participation. A shift in focus in research and clinical practice is required to move from individual impairments and capacities, distinguished from environmental barriers and enablers, to a more fluid construct that reflects the transactional nature of the person-activity-environment interfaces [43]. Adopting multi-dimensional and transactional models will be essential to support an understanding of the organisational and emotional issues presenting in DCD.

## References

[1] American Psychiatric Association (2013). Diagnostic and statistical manual of mental disorders (DSM-5®).

[2] Bodison SC (2015). Developmental dyspraxia and the play skills of children with autism. Am J Occup Ther.

[3] MacNeil LK, Mostofsky SH (2012). Specificity of dyspraxia in children with autism. Neuropsychol.

[4] McAuliffe D, Pillai AS, Tiedemann A, Mostofsky SH, Ewen JB (2017). Dyspraxia in ASD: impaired coordination of movement elements. Autism Res.

[5] Miller M, Chukoskie L, Zinni M, Townsend J, Trauner D (2014). Dyspraxia, motor function and visual–motor integration in autism. Beh Brain Res.

[6] Blank R, Barnett A, Cairney J, Green D, Kirby A, Polatajko H, Rosenblum S, Smits-Engelsman B, Sugden D, Vinçon S, Wilson P: International Clinical Practice Guideline on the definition, diagnosis, assessment, intervention and psycho-social aspects of Developmental Coordination Disorder (DCD). In Preparation: Euro Aca Child Disabil. 10.1111/dmcn.14132 PMC6850610 30671947

[7] Blank R, Smits-Engelsman BO, Polatajko H, Wilson P (2012). European Academy for Childhood Disability (EACD): recommendations on the definition, diagnosis and intervention of developmental coordination disorder (long version). Dev Med Child Neurol.

[8] Lingam RP, Novak C, Emond A, Coad JE (2014). The importance of identity and empowerment to teenagers with developmental co-ordination disorder. Child Care Health Dev.

[9] Cairney J, Rigoli D, Piek J (2013). Developmental coordination disorder and internalizing problems in children: the environmental stress hypothesis elaborated. Dev Rev.

[10] Goulardins JB, Rigoli D, Licari M, Piek JP, Hasue RH, Oosterlaan J, Oliveira JA (2015). Attention deficit hyperactivity disorder and developmental coordination disorder: two separate disorders or do they share a common etiology. Beh brain res.

[11] Green D, Baird G, Sugden D (2006). A pilot study of psychopathology in developmental coordination disorder. Child Care Health Dev.

[12] Harrowell I, Hollén L, Lingam R, Emond A (2017). Mental health outcomes of developmental coordination disorder in late adolescence. Dev Med Child Neurol.

[13] Sigurdsson E, Van Os J, Fombonne E (2002). Are impaired childhood motor skills a risk factor for adolescent anxiety? Results from the 1958 UK birth cohort and the National Child Development Study. Am J Psych.

[14] Hellgren L, Gillberg CI, Bågenholm A, Gillberg C (1994). Children with deficits in attention, motor control and perception (DAMP) almost grown up: psychiatric and personality disorders at age 16 years. J Child Psychol Psych.

[15] Kirby A, Sugden D, Edwards L (2010). Developmental co-ordination disorder (DCD): more than just a movement difficulty. J Res Spec Educ Needs.

[16] Wilmut K, Du W, Barnett AL (2015). How do i fit through that gap? Navigation through aperturein adults with and without developmental coordination disorder. PLoS One.

[17] Kirby A, Edwards L, Sugden D, Rosenblum S (2010). The development and standardization of the adult developmental co-ordination disorders/dyspraxia checklist (ADC). Res Dev Disabil.

[18] van Der Linde BW, van Netten JJ, Otten BE, Postema K, Geuze RH, Schoemaker MM (2014). Psychometric properties of the DCDDaily-Q: a new parental questionnaire on children’s performance in activities of daily living. Res Dev Disabil.

[19] Dunford C, Bannigan K, Rathmell S (2016). Learning to ride a bike: developing a therapeutic intervention. CYPF Journal.

[20] Kirby A, Williams N, Thomas M, Hill EL (2013). Self-reported mood, general health, wellbeing and employment status in adults with suspected DCD. Res Dev Disabil.

[21] de Oliveira RF, Wann JP (2012). Driving skills of young adults with developmental coordination disorder: maintaining control and avoiding hazards. Hum Mov Sci.

[22] Payne S. How is life experienced by teenagers with dyspraxia? An interpretative phenomenological analysis. Coventry University: Unpublished Doctoral dissertation.

[23] Cocks N, Barton B, Donelly M (2009). Self-concept of boys with developmental coordination disorder. Phys Occup Ther Ped.

[24] Engel-Yeger B, Hanna KA (2010). The relationship between developmental co-ordination disorders, child’s perceived self-efficacy and preference to participate in daily activities. Child Care Health Dev.

[25] Lingam R, Jongmans MJ, Ellis M, Hunt LP, Golding J, Emond A (2012). Mental health difficulties in children with developmental coordination disorder. Peds.

[26] Gagnon-Roy M, Jasmin E, Camden C (2016). Social participation of teenagers and young adults with developmental co-ordination disorder and strategies that could help them: results from a scoping review. Child Care Health Dev.

[27] Hill EL, Brown D (2013). Mood impairments in adults previously diagnosed with developmental coordination disorder. J Mental Health.

[28] Saban MT, Ornoy A, Parush S (2014). Executive function and attention in young adults with and without developmental coordination disorder–a comparative study. Res Dev Disabil.

[29] Campbell WN, Missiuna C, Vaillancourt T (2012). Peer victimization and depression in children with and without motor coordination difficulties. Psychol Sch.

[30] Pratt ML, Hill EL (2011). Anxiety profiles in children with and without developmental coordination disorder. Res Dev Disabil.

[31] Pearsall-Jones JG, Piek JP, Rigoli D, Martin NC, Levy F (2011). Motor disorder and anxious and depressive symptomatology: a monozygotic co-twin control approach. Res Dev Disabil.

[32] Jongmans M, Dubowitz L, Demetre JD, Henderson SE (1996). How local is the impact of a specific learning difficulty on premature children’s evaluation of their own competence?. J Child Psychol Psych.

[33] Jarus T, Lourie-Gelberg Y, Engel-Yeger B, Bart O (2011). Participation patterns of school-aged children with and without DCD. Res Dev Disabil.

[34] Barnett AL, Dawes H, Wilmut K (2013). Constraints and facilitators to participation in physical activity in teenagers with developmental co-ordination disorder: an exploratory interview study. Child Care Health Dev.

[35] Poulsen AA, Ziviani JM, Cuskelly M (2008). Leisure time physical activity energy expenditure in boys with developmental coordination disorder: the role of peer relations self-concept perceptions. OTJR: Occup Part Health.

[36] Biddle SJ, Asare M (2011). Physical activity and mental health in children and adolescents: a review of reviews. Brit J Sports Med.

[37] Payne S, Ward G, Turner A, Taylor MC, Bark C (2013). The social impact of living with developmental coordination disorder as a 13-year-old. Brit J Occup Ther.

[38] McCarthy L. Dyspraxia—“Is it a battle of the sexes?”. UK: Dyspraxia Foundation Retrieved from: https://dyspraxiafoundation.org.uk/dyspraxia-is-battle-sexes/

[39] Cairney J, Hay J, Veldhuizen S, Faught BE (2010). Trajectories of cardiorespiratory fitness in children with and without developmental coordination disorder: a longitudinal analysis. Brit J Sport Med.

[40] Bejerot S, Humble MB (2013). Childhood clumsiness and peer victimization: a case–control study of psychiatric patients. BMC Psychiatry.

[41] Morton J (2008). Understanding developmental disorders: a causal modelling approach.

[42] Green D, Chambers ME, Sugden DA (2008). Does subtype of developmental coordination disorder count: is there a differential effect on outcome following intervention?. Hum Mov Sci.

[43] King G, Imms C, Stewart D, Freeman M, Nguyen T (2017). A transactional framework for pediatric rehabilitation: shifting the focus to situated contexts, transactional processes, and adaptive developmental outcomes. Disabil Rehabil.

[44] Green D (2017). Time and relativity in therapeutic rehabilitation. Dev Med Child Neurol.

[45] Albrecht EC, Khetani MA (2017). Environmental impact on young children’s participation in home-based activities. Dev Med Child Neurol.

[46] Piek JP, Barrett NC, Allen LS, Jones A, Louise M (2005). The relationship between bullying and self-worth in children with movement coordination problems. Br J Educ Psychol.

[47] Mancini VO, Rigoli D, Cairney J, Roberts LD, Piek JP (2016). The elaborated environmental stress hypothesis as a framework for understanding the association between motor skills and internalizing problems: a mini-review. Front Psychol.

[48] Lingam R, Hunt L, Golding J, Jongmans M, Emond A (2009). Prevalence of developmental coordination disorder using the DSM-IV at 7 years of age: a UK population–based study. Pediatrics.

[49] Tal-Saban M, Zarka S, Grotto I, Ornoy A, Parush S (2012). The functional profile of young adults with suspected developmental coordination disorder (DCD). Res Dev Disabil.

[50] Schott N, El-Rajab I, Klotzbier T (2016). Cognitive-motor interference during fine and gross motor tasks in children with developmental coordination disorder (DCD). Res Dev Disabil.

[51] Ruddock S, Piek J, Sugden D, Morris S, Hyde C, Caeyenberghs K, Wilson P (2015). Coupling online control and inhibitory systems in children with developmental coordination disorder: goal-directed reaching. Res Dev Disabil.

[52] Pratt ML, Leonard HC, Adeyinka H, Hill EL (2014). The effect of motor load on planning and inhibition in developmental coordination disorder. Res Dev Disabil.

[53] Wilson PH, Smits-Engelsman B, Caeyenberghs K, Steenbergen B, Sugden D, Clark J, Mumford N, Blank R (2017). Cognitive and neuroimaging findings in developmental coordination disorder: new insights from a systematic review of recent research. Dev Med Child Neurol.

[54] Langevin LM, MacMaster FP, Dewey D (2015). Distinct patterns of cortical thinning in concurrent motor and attention disorders. Dev Med Child Neurol.

[55] Frith CD, Blakemore SJ, Wolpert DM (2000). Abnormalities in the awareness and control of action. Philos Trans R Soc Lond B.

[56] Mosconi MW, Mohanty S, Greene RK, Cook EH, Vaillancourt DE, Sweeney JA (2015). Feedforward and feedback motor control abnormalities implicate cerebellar dysfunctions in autism spectrum disorder. J Neurosci.

[57] Vogt S, Rienzo FD, Collet C, Collins A, Guillot A (2013). Multiple roles of motor imagery during action observation. Front Hum Neurosci.

[58] Debrabant J, Vingerhoets G, Van Waelvelde H, Leemans A, Taymans T, Caeyenberghs K (2016). Brain connectomics of visual-motor deficits in children with developmental coordination disorder. J Pediatr.

[59] Zwicker JG, Missiuna C, Harris SR, Boyd LA (2012). Developmental coordination disorder: a pilot diffusion tensor imaging study. Pediatr Neurol.

[60] Reynolds JE, Licari MK, Billington J, Chen Y, Aziz-Zadeh L, Werner J, Winsor AM, Bynevelt M (2015). Mirror neuron activation in children with developmental coordination disorder: a functional MRI study. Int J Dev Neurosci.

[61] Green D, Arscott C, Barnett AL, Henderson L, Henderson SE, Huber J, May A, Baird G. Impairment of movement and social difficulties in children with Autism Spectrum Disorder (Asperger Syndrome) and Developmental Coordination Disorder-how are they perceived by the children and their teachers? in Barnett AL, Sugden DA, editors. Moving, Developing and Learning. A Festschrift in celebration of the career of Sheila E. Henderson. Oxford Brookes University: 2015. ISBN: 978-1-873640-87-6.

[62] Debrabant J, Gheysen F, Caeyenberghs K, Van Waelvelde H, Vingerhoets G (2013). Neural underpinnings of impaired predictive motor timing in children with developmental coordination disorder. Res Dev Disabil.

[63] Bonthrone AF, Clark CA, Morgan A, Green D, Liegeois F. Subcortical correlates of receptive language impairment in children aged 8-10 with developmental coordination disorder. Soc. Neurobiology of Language Conference Proceedings 2016:47. Available from: http://www.neurolang.org/programs/SNL_2016_Abstracts_Download.pdf.

[64] Weinstein M, Green D, Rudisch J, Zielinski IM, Benthem-Muñiz M, Jongsma ML, et al. Understanding the relationship between brain and upper limb function in children with unilateral motor impairments: a multimodal approach. Euro J Paediatr Neurol. 2017; 10.1016/j.ejpn.2017.09.012. 10.1016/j.ejpn.2017.09.012 29111113

[65] Paulesu E, Frith U, Snowling M, Gallagher A, Morton J, Frackowiak RS, Frith CD (1996). Is developmental dyslexia a disconnection syndrome? Evidence from PET scanning. Brain.

[66] Dancza K, Missiuna C, Pollock N (2017). Occupation-centred practice: when the classroom is your client. In Rodger S, Kenney/Behr, editors. Occupation-centred practice with children: a practical guide for occupational therapists.

[67] Graham F, Rodger S, Kennedy-Behr A, Rodger S, Kenney/Behr (2017). Occupational performance coaching (OPC): enabling caregiver’s and childrens’ occupational performance. Occupation-Centred practice with children: a practical guide for occupational therapists.

[68] Anaby D, Mercerat C, Tremblay S (2017). Enhancing youth participation using the PREP intervention: parents’ perspectives. Int J Environ Res Public Health.

[69] Green D, Martini R: Group approaches in childhood. In Dawson D, McEwen S, Polatajko H, editors. Enabling participation across the lifespan: advancements, adaptations and extensions of the CO-OP approach. Washington: Amer OT Assoc Pubs.

[70] Kramer JM, Roemer K, Liljenquist K, Shin J, Hart S (2014). Formative evaluation of project TEAM (teens making environment and activity modifications). Intellect Dev Disabil.

